# Unraveling the Bioactive Profile, Antioxidant and DNA Damage Protection Potential of Rye (*Secale cereale*) Flour

**DOI:** 10.3390/antiox10081214

**Published:** 2021-07-28

**Authors:** Pinderpal Kaur, Kawaljit Singh Sandhu, Sneh Punia Bangar, Sukhvinder Singh Purewal, Maninder Kaur, Rushdan Ahmad Ilyas, Muhammad Rizal Muhammad Asyraf, Muhammad Rizal Razman

**Affiliations:** 1 Department of Food Science and Technology, Maharaja Ranjit Singh Punjab Technical University, Bathinda 151001, India; pinderpal94@gmail.com (P.K.); purewal.0029@gmail.com (S.S.P.); 2 Department of Food, Nutrition and Packaging Sciences, Clemson University, Clemson, SC 29634, USA; 3 Department of Food Science and Technology, Guru Nanak Dev University, Amritsar 143005, India; mandyvirk@rediffmail.com; 4 School of Chemical and Energy Engineering, Faculty of Engineering, Universiti Teknologi Malaysia (UTM), Johor Bahru 81310, Malaysia; ahmadilyas@utm.my; 5 Centre for Advanced Composite Materials (CACM), Universiti Teknologi Malaysia (UTM), Johor Bahru 81310, Malaysia; 6 Department of Aerospace Engineering, Faculty of Engineering, Universiti Putra Malaysia (UPM), Serdang 43400, Malaysia; asyrafriz96@gmail.com; 7 Research Centre for Sustainability Science and Governance (SGK), Institute for Environment and Development (LESTARI), Universiti Kebangsaan Malaysia (UKM), Bangi 43600, Malaysia

**Keywords:** rye cultivars, extraction, phytochemicals, total phenolic compounds, antioxidant properties

## Abstract

Six different solvents were used as extraction medium (water, methanol, ethanol, acidified methanol, benzene and acetone) to check their phenolics extraction efficacy from flour of two rye cultivars. Rye extracts with different solvents were further analyzed for the estimation of phytochemicals and antioxidant properties. Different tests (TPC, TAC, DPPH, FRAP, ABTS, RPA and CTC) were performed to check the antioxidant properties and tannin contents in extracts. A bioactive profile of a rye cultivar indicated the presence of total phenolic compounds (0.08–2.62 mg GAE/g), total antioxidant capacity (0.9–6.8 mg AAE/g) and condensed tannin content (4.24–9.28 mg CE/100 g). HPLC was done to check phenolics in rye extract with the best solvent (water), which indicated the presence of Catechol (91.1–120.4 mg/100 g), resorcinol (52–70.3 mg/100 g), vanillin (1.3–5.5 mg/100 g), ferulic acid (1.4–1.5 mg/100 g), quercetin (4.6–4.67 mg/100 g) and benzoic acid (5.3 mg/100 g) in rye extracts. The presence of DNA damage protection potential in rye extracts indicates its medicinal importance. Rye flour could be utilized in the preparation of antioxidant-rich health-benefiting food products.

## 1. Introduction

Frequent use of unhealthy food products (junk foods), less physical activity, a busy working schedule and deficiency of health-benefiting nutrients in a daily diet may heighten susceptibility to chronic diseases [1,2]. The addition of whole grains and grain-based healthy food products are favorably supported in diet charts, as they provide significant amounts of protein, fibers, carbohydrates, minerals and bioactive compounds. Rye (Secale cereale) is considered an important traditional cereal crop, and is cultivated worldwide. Rye stands in second place as a cereal grain after wheat, whose flour is utilized for the preparation of bakery products, especially bread and biscuits [3]. Rye (*Secale cereale*) belongs to the *Poaceae* family and its genus is Secale. Rye crops are believed to have originated in southwestern Asia and are widely cultivated in Europe, North America and Asia [4]. Rye crops are well known for their adaptability toward harsher environmental conditions. The crops can germinate well at 1.11–3.33 °C. Rye grains are oval/wedge shaped and light to dark brownish in color. Rye grains are genetically related to wheat and barley grains [5]. However, the major difference lies in the size of grains, their nutritional composition and the organoleptic properties they possess. For the proper growth of rye grains, the preferred soil types are light loam/sandy, and well drained/fairly dry soil with a pH ranging from 4.5–8.2. Rye can grow in sandy soils with less nutrients than other soil types. Rye grains are capable of tolerating saline conditions, a low pH and a high concentration of aluminum. Being a long-day plant, rye crops require 40–60 days to shift in their reproductive stage. The vegetation period for rye grains may vary from 120–150 days.

Rye is considered a stress-tolerant and disease-resistant cereal crop [6]. Rye grains have been scrutinized as a good source of fiber, and contain proteins, minerals and bioactive phenolic compounds that have been well documented for their health-benefiting effects [7,8,9,10,11]. Hundreds of bioactive phytochemicals from the extracts of natural resources have been identified and being studied for their health-benefiting antioxidant properties [10,12,13,14]. Liu et al. [15] demonstrated that health-benefiting properties of food products are mainly due to the syringic and additive effects of bioactive compounds. Cereal grains and their milling fractions may possess mixtures of bioactive compounds. The bioactive profile of rye grains and milling fractions indicates the presence of ferulic acid, sinapic acid, p-Coumaric acid, syringic acid and vanillic acid, followed by p-hydroxybenzoic acid [16,17,18,19]. The unique characteristic feature of these specific phytochemicals is their solvent specificity, as some of them are soluble in an aqueous phase whereas other are either soluble in organic solvents or a combination of aqueous and organic phases [20,21,22,23,24,25,26,27,28,29]. Scantiness of scientific information regarding the effect of different solvent types on rye phenolics incited us to design the present work. The objective of the present research work is to analyze the effect of different solvents on rye phenolics, screening for specific bioactive compounds and their quantifications using HPLC analysis.

## 2. Materials and Methods

Rye cultivars (black and white) were collected (IARI, 28.08° N; 77.12° E; 228.61 m) from PUSA, New Delhi. Grains were washed well with tap water, oven dried (Rescholar, India) at 45 °C for 48 h and stored in cross-zip airtight pouches/plastic containers for further experimental work.

### 2.1. Chemicals and Glassware’s

Solvents (ethanol, acetone, benzene, methanol and HCl, etc.) and chemicals (DPPH (C_18_H_12_N_5_O_6_); potassium persulphate (K_2_S_2_O_8_), Folin-Ciocalteu (C_10_H_5_NaO_5_S) reagent (FCR); disodium hydrogen orthophosphate, trichloroacetic acid (C_2_HCl_3_O_2_), ferric chloride (FeCl_3_), potassium ferricyanide (C_6_N_6_FeK_3_), sodium carbonate (Na_2_CO_3_), ammonium molybdate, 2,2′-azino-bis[3-ethylbenzothiazoline-6-sulfonate] (ABTS), etc.) were of either HPLC or analytic grade (Sigma Aldrich, HiMedia, India). Standards (HPLC grade) used during different antioxidant assays were procured from Sigma Aldrich. HPLC grade standards (Resorcinol, catechol, vanillin, ferulic acid, quercetin, benzoic acid, ascorbic acid, catechin, gallic acid and p-Coumaric acid) were procured from HiMedia and Sigma-Aldrich, respectively.

### 2.2. Sample Preparation

**Figure 1:**
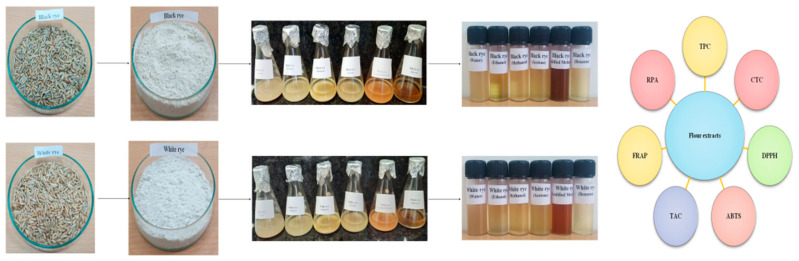
Sample preparation and type of analysis performed.

Rye grains from different cultivars were milled to fine flour (Bajaj, India) and passed through a sieve (60BSS) to obtain a uniform size of flour particles (250 micron). Defattation of different rye flour samples (black and white) was carried out with Hexane (1:4 *w*/*v*; 10 min; thrice). Defatted samples were dried in a hot air oven (40 °C for 15–24 h, Rescholar, India) and stored (deep freezer, −20 °C, Vestfrost, India). Thereafter, defatted samples were processed using absolute benzene, ethanol, acetone, methanol, acidified methanol (HCl-Methanol; 1:99 *v*/*v*) and water in ratio of 1:10 *w*/*v* at 50 °C for 40 min in a water bath (Rescholar, India). The flour–solvent mixture was filtered (Whatman No. 1 filter paper 100125R, HiMedia) and extracts were stored in sample-storing vials at 4 °C in a refrigerator (Samsung, India). Rye flour extracts prepared in different solvents were named water rye extract (WRE), ethanol rye extract (ERE), methanol rye extract (MRE), acetone rye extract (ARE), acidified methanol rye extract (AMRE) and benzene rye extract (BRE), respectively. The entire process is represented in Figure 1.

### 2.3. Phytochemical Analysis

Rye extracts (WRE, ERE, MRE, ARE, AMRE and BRE) were screened for the presence of various phytochemicals [30].

### 2.4. Total Phenolic Content (TPC) and HPLC Analysis

TPC in rye extracts (WRE, ERE, MRE, ARE, AMRE and BRE) were analyzed using the FCR (Folin-Ciocalteu reagent) method [31]. FCR (500 µL) was added in an aliquot (100 µL) of each rye extract (WRE, ERE, MRE, ARE, AMRE and BRE) followed by an aqueous sodium carbonate solution addition (1500 µL, 20%). The resulting reaction mixture (extracts and reagents) was kept undisturbed (20 min) in the dark at ambient temperature. After 20 min, distilled water was added to prepare the final volume up to a mark (10 mL). Absorbance of blue colored extract–reagent mixture was recorded (765 nm; Shimadzu, India). Gallic acid (mg/mL stock, HiMedia) was used (standard). Results were expressed as mg GAE (gallic acid equivalent) g^−1^ dry weight basis (dwb).

Water extracts (BR and WR) were screened through qualitative and quantitative HPLC analysis ((Shimadzu 10 AVP HPLC system) for specific bioactive compounds. HPLC grade standards (Resorcinol, catechol, vanillin, ferulic acid, quercetin, benzoic acid, ascorbic acid, catechin, gallic acid and p-Coumaric acid) were used. Four different phases were used: phase-A (methanol, HPLC grade), phase-B (glacial acetic acid, 0.1%), phase-C (ortho-phosphoric acid, 0.1%) and phase-D (Acetonitrile, HPLC grade). The ratio of optimized gradient phase during the analysis was 30:30:25:15 with a flow rate of 1.2 mL/min and a temperature of 30 °C.

### 2.5. DPPH (C_18_H_12_N_5_O_6_) Assay

Percent (%) inhibition of DPPH reagent by rye extracts (WRE, ERE, MRE, ARE, AMRE and BRE) was measured [32]. An aliquot (100 μL) of rye extracts (WRE, ERE, MRE, ARE, AMRE and BRE) was added in to the testing vial (5 mL) followed by a DPPH reagent addition (3 mL). The resulting DPPH–extract mixture was then kept undisturbed (30 min). The changes in absorbance (517 nm) were noted.

Percent (%) DPPH inhibition: A (Extract_t=0_) − A (Extract_t=30_)/A (Extract_t=0_) × 100

### 2.6. ABTS Assay

Percent (%) inhibition of ABTS reagent by rye extract (WRE, ERE, MRE, ARE, AMRE and BRE) was measured [33]. An aliquot (100 μL) of rye extracts (WRE, ERE, MRE, ARE; AMRE and BRE) was added into a testing vial (5 mL) followed by an ABTS reagent addition (3 mL). The resulting ABTS–extract mixture was then kept undisturbed (10 min). The changes in absorbance (732 nm) were noted.

Percent (%) ABTS inhibition: A (Extract_t=0_) − A (Extract_t=10_)/A (Extract_t=0_) × 100

### 2.7. Total Antioxidant Capacity (TAC)

The TAC of rye extracts (WRE, ERE, MRE, ARE, AMRE and BRE) was determined [34]. The standard used during the TAC assay was ascorbic acid. A reagent was prepared to evaluate the antioxidant properties in rye extracts using ammonium molybdate (4 mM), conc. H_2_SO_4_ (0.6 M) and sodium hydrogen orthophosphate (28 mM). Rye extracts (100 μL) were allowed to react with the TAC reagent (3 mL). The resulting TAC reagent–extract mixture was warmed (95 °C/90 min). Ascorbic acid (mg/mL) was used as the standard. The TAC value of rye extracts was calculated from the equation generated, and results were expressed as mg AAE/g. Absorbance of the rye extract–reagent mixture and standard solution was recorded (695 nm).

### 2.8. Reducing Power Activity (RPA)

The RPA of rye extracts (WRE, ERE, MRE, ARE, AMRE and BRE) was determined [35]. Rye extracts (100 μL) were allowed to react with an aqueous potassium ferricyanide (K_3_[Fe(CN)_6_]) solution (1%, 100 μL) followed by incubation in a water bath (50 °C/30 min). The reaction mixture was cooled at room temperature followed by addition of trichloroacetic acid (1% 100 μL) and aqueous ferric chloride solutions (0.1%, 100 μL). The colored mixture was incubated (15 min), which was diluted with distilled water after incubation to prepare the final volume (10 mL). Absorbance of green colored complex was noted (700 nm). The standard used during the RPA assay was Quercetin. The RPA was calculated from the equation generated and results were expressed as mg QE/g. Absorbance of the extract–reagent mixture and standard solution was recorded (700 nm).

### 2.9. Ferric Reducing Antioxidant Power (FRAP)

The FRAP of rye extracts (WRE, ERE, MRE, ARE, AMRE and BRE) was determined [36]. A stock solution for FRAP reagent was prepared (TPTZ (2,4,6-tripyridyl-s-triazine) (10 mM) solution in HCl (40 mM), acetate buffer (300 mM) and FeCl_3_·6H_2_O (20 mM)). The fresh solution was prepared by mixing the TPTZ solution, acetate buffer and FeCl_3_·6H_2_O solution at a ratio of 1:10:1, then heating in a water bath at 45 °C before performing the assay. Rye extracts (100 µL) were taken in sample vials to react with the FRAP reagent (3 mL, 10 min under darkness). Reading of the rye extract–reagent mixture (blue colored ferrous tripyridyltriazine complex) was noted (595 nm).

### 2.10. Condensed Tannin Content (CTC)

The CTC of rye extracts (WRE, ERE, MRE, ARE, AMRE and BRE) was assessed using the Vanillin (C_8_H_8_O_3_)–HCl method [37]. Rye extracts (100 μL) were allowed to react with Vanillin–HCl (1:0.5 *v*/*v*). The reaction mixture was kept undisturbed at room temperature (15 min). Absorbance of the extract–reagent mixture against a blank was recorded (500 nm). For the preparation of the standard curve, catechin was used as the standard. CTC in rye extracts was expressed as mg CE (catechin equivalent)/100 g.

### 2.11. DNA Damage Protection Potential (DDPPA) Assay

WRE (black and white rye) was used during DDPPA, and the assay was performed as per previously reported conditions [38]. Different reagents were used (1:1:1 *v*/*v*) to prepare Fenton’s reagent (ascorbic acid (500 μM), Ferric chloride (800 μM) and Hydrogen peroxide (30 mM)). The mixture used to initiate the reaction mechanism was DNA (2.5 μL), nuclease free double distilled water (2.5 μL), extract (5.0 μL) and Fenton’s reagent (10 μL). Reaction mixture was then incubated in water bath (37 °C/45 min). After incubation, loading buffer (2.5 μL; 0.25% Bromophenol blue and glycerol 50%) was added to the reaction mixture. The results were noted on Agarose gel electrophoresis (1.0%) stained with ethidium bromide (4 μL). The DNA protection potential of rye extracts were evaluated using the retention percentage of the normalized super-coiled DNA, as given below.

$$\mathrm{DNA}\;\mathrm{retention}\;\left(\%\right)=\frac{\mathrm{Intensity}\;\mathrm{of}\;\mathrm{supercoiled}\;\mathrm{DNA}\;\mathrm{with}\;\mathrm{the}\;\mathrm{oxidative}\;\mathrm{radical}\;\mathrm{and}\;\mathrm{extract}}{\mathrm{Intensity}\;\mathrm{of}\;\mathrm{supercoiled}\;\mathrm{DNA}\;\left(\mathrm{control}\right)}\times 100$$

### 2.12. Statistical Analysis

Experiments were performed in triplicate to generate results. Afterwards, mean value and standard deviation were calculated. Tukey’s test was used to test significant differences among experiments. Differences among means were considered statistically significant at a 5% level. The score plot, loading plot and correlation for determining the relationship was generated using Minitab software 18.

## 3. Results and Discussion

### 3.1. Bioactive Compounds

Rye extracts (BR and WR) were subjected to prelim phytochemical assessment, with the results reported in Table 1. The WRE showed the presence of coumarins, tannins, sugar, saponins and protein, whereas the ERE and ARE showed only saponins. Further, BRE showed the presence of flavonoids, protein and saponins, followed by the MRE, which showed only two compounds (i.e., coumarins and saponins). The AMRE indicated the presence of coumarins, flavonoids, sugars, tannins and saponins.

**Figure 2:**
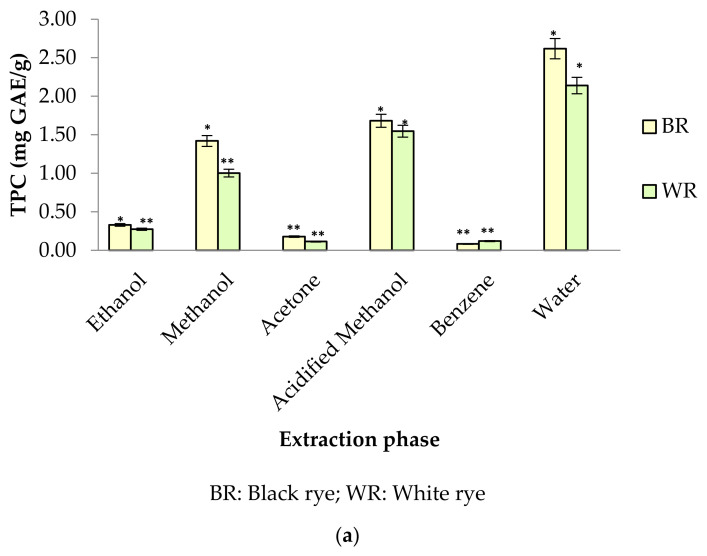

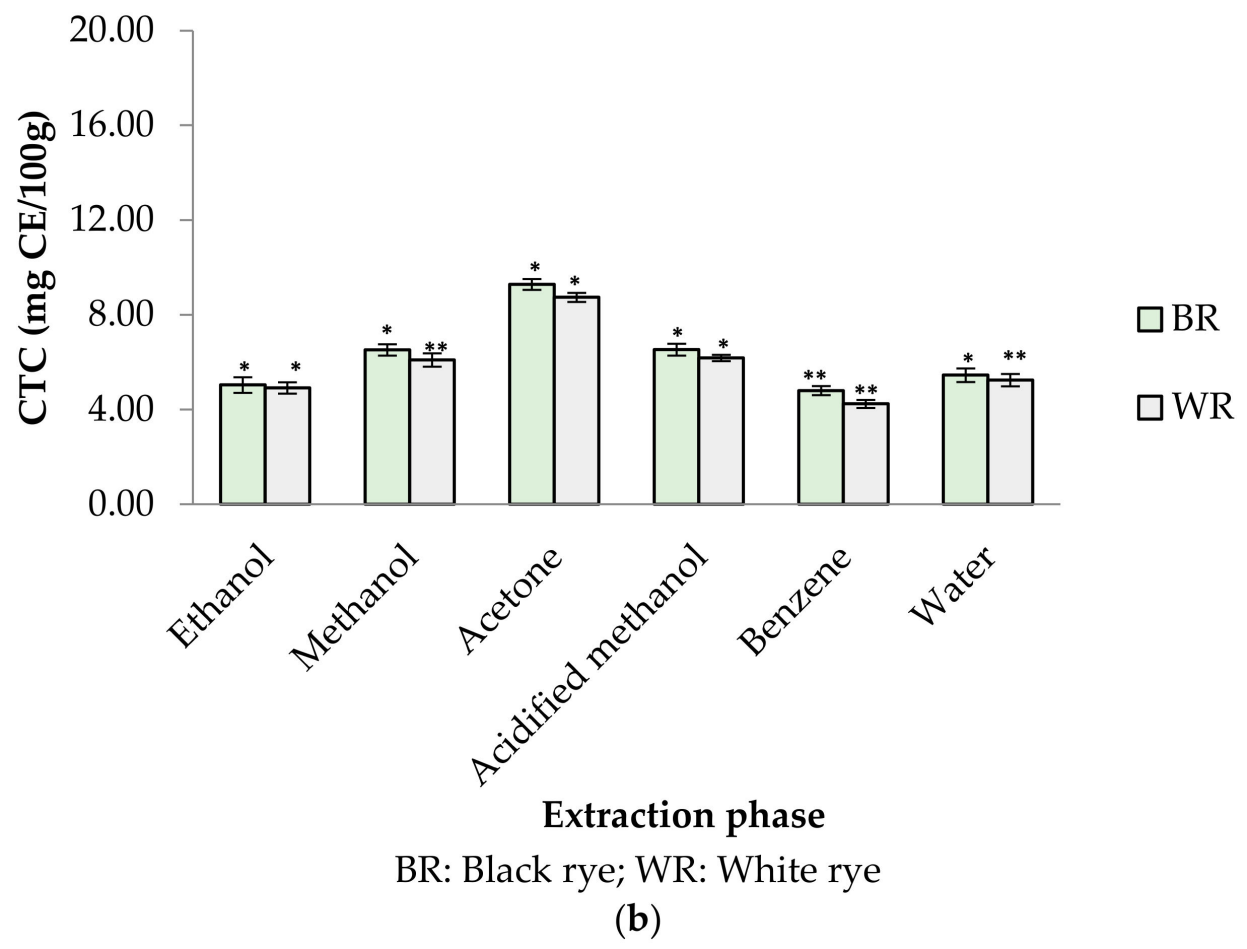
(**a**) TPC in different rye extracts; (**b**) CTC in different rye extracts. * significantly different at *p* < 0.05, ** significantly different at *p* < 0.01.

Phenolic profiles of substrates like fruits/vegetables and cereal grains are gaining more attention among researchers, industries and consumers because of their significance to human health. In the present study, six different extraction mediums (ethanol, acetone, methanol, acidified methanol, water and benzene) were used to extract out phenolic compounds from rye cultivars (BR and WR). Among the selected rye cultivars, black rye (BR) possessed a higher amount of phenolic compounds in the different extraction mediums than the white rye (WR). Extraction mediums used during the experimental work of the present study played an important role when extracting phenolics. Water proved to be the better solvent for extracting total phenolics (TPC) from rye flour as compared to acidified methanol, methanol, ethanol, acetone and benzene. The highest TPC was observed in water extracts (BR (2.62 mg GAE/g); WR (2.14 mg GAE/g)), followed by acidified methanol extracts (BR (1.68 mg GAE/g); WR (1.55 mg GAE/g)), methanol (BR (1.42 mg GAE/g); WR (1.00 mg GAE/g)) and ethanol (BR (0.33 mg GAE/g); WR (0.27 mg GAE/g)). As compared to water, neither acidified methanol, methanol, ethanol, acetone nor benzene were efficient enough to extract out a significant amount of phenolics from rye flour (Figure 2a). TPC in water rye extracts (WRE) of BR (2.62 mg GAE/g) and WR (2.14 mg GAE/g) was in consistent with the results of Mishra et al. [39], who reported a maximum TPC of 2.19 mg/g in water extracts of rye. The TPC (1–1.42 mg GAE/g) in the MREs (BR, WR) agreed with the finding of Zielinski et al. [40], who reported 1.35–1.47 mg/g TPC in 80% methanolic extracts. Total phenolic content in methanolic (80%) extracts of rye varied from 0.98–3.36 mg GAE/g in reports by Kulichova et al. [12] and Ragaee et al. [41]. Michalska et al. [17] reported the effect of phosphate buffered saline (PBS) and methanol (80%) on recovery of phenolics from rye grains. The amount of phenolics was 2.31 mg/g for rye extracts prepared in PBS and 1.43 mg/g for methanolic extract. The CTC in BR and WR extracts was observed to be 4.24–9.28 mg CE/100 g (Figure 2b). The highest CTC was noted in acetone extracts of BR (9.28 mg CE/100 g) and WR (8.74 mg CE/100 g), whereas the lowest amount was noted in benzene extracts of BR (4.80 mg CE/100 g) and WR (4.24 mg CE/100 g).

### 3.2. Phenolics in Rye Extracts

**Figure 3:**
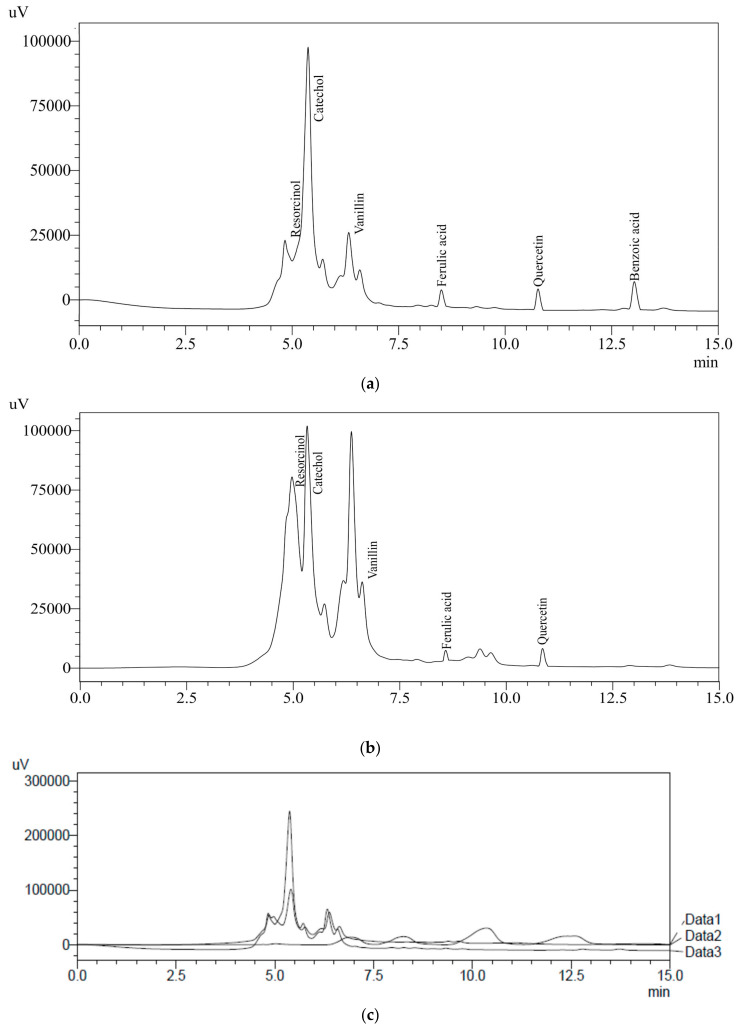
(**a**) HPLC chromatograph of BRE; (**b**) HPLC chromatograph of WRE; (**c**) overlay of HPLC standards with BRE and WRE.

Water extract (WRE) of BR and WR possessing higher amounts of TPC were analyzed using HPLC to identify and quantify the specific phenolic compounds. Ten different HPLC grade standards (Resorcinol, catechol, catechin, cinnamic acid, ascorbic acid, benzoic acid, ferulic acid, gallic acid, vanillin and quercetin) were used for the screening of specific compounds in rye extracts. WRE of black cultivar (BR) showed the presence of resorcinol (70.4 mg/100 g), catechol (120.4 mg/100 g), vanillin (5.5 mg/100 g), ferulic acid (1.52 mg/100 g), quercetin (4.68 mg/100 g) and benzoic acid (5.3 mg/100 g) (Figure 3a and Table 2). HPLC analysis of white cultivar (WR) WRE indicated resorcinol (52 mg/100 g), catechol (91.1 mg/100 g), vanillin (1.4 mg/100 g), ferulic acid (1.46 mg/100 g) and quercetin (4.6 mg/100 g) (Figure 3b and Table 2). An overlay of standards along with chromatograms of the rye extracts is presented in Figure 3c. Earlier published reports have also indicated the presence of ferulic acid, benzoic acid and vanillin in rye grains [12,39,42]. Ferulic acid content in BR and WR was comparable with the results (0.61–65.74 mg/100 g) reported by Pihlava et al. [42]; however, it was significantly higher than the results (0.438–0.855 mg/100 g) reported by Mishra et al. [39] in water extracts. Vanillin and ferulic acid content observed by Kulichova et al. [12] was lower (0.075–0.3188 and 0.1903–0.6227 mg/100 g, respectively) as compared to the results of current study. Benzoic acid content BR cultivar was higher in the present study that that (0.077–0.508 mg/100 g) reported by Mishra et al. [39]. Seasonal variation, soil conditions, cultivar type, agricultural practice and storage conditions have significant effects on the bioactive profiles of natural resources [43,44,45,46]. These factors play a vital role in analyzing the concentrations of bioactive compounds in different studies. Previous reports on rye grains have indicated the presence of ferulic acid (86–117 mg/100 g), caffeic acid (0.42–1 mg/100 g), p-Coumaric acid (0.35–6.5 mg/100 g), sinapic acid (0.23–130 mg/100 g), vanillic acid (0.25–3 mg/100 g) and p-hydroxybenzoic acid (0.68–0.97 mg/100 g) [10,16,42,47,48,49,50]. Further, the presence of specific bioactive metabolites in rye extracts makes rye a healthier food choice. The health-benefiting nature of bioactive compounds present in rye extracts are reported in Table 3.

### 3.3. Antioxidant Properties

Different extracts of rye (WRE, ERE, MRE, ARE, AMRE and BRE) were assessed using TAC, DPPH, RPA, ABTS and FRAP assays. These antioxidant assays are either specific color-forming or decolorizing tests that have been widely adopted for screening antioxidant properties in natural extracts via free radical scavenging [2,20,51]. Antioxidant properties of rye extracts are reported in Table 4. DPPH activity is expressed as percent inhibition, indicated by the ability of an extract to decolorize and convert a purple-colored DPPH reagent to mustard yellow (stabilized form) [58,59,60]. Among extracts (WRE, ERE, MRE, ARE, AMRE and BRE) of selected rye cultivars (BR and WR), WRE showed maximum activity against DPPH at 84% for the BR and 80.3% for the WR. Percent inhibition activity against DPPH radical was observed to be 64.8–74.3% for the AMRE, followed by 38.7–43.5% for the MRE, 21.3–31.4% for the ERE, 14.8–26% for the ARE and 10.7–14.1% for the BRE. Further, another antioxidant assay ABTS was performed to check the percent inhibition activity in rye extracts. Percent (%) inhibition against ABTS was also observed to be higher in water extracts of both rye cultivars: 89.7% for black and 87.6% for white. Rye extracts (AMRE, MRE, ERE, ARE and BRE) other than water also possessed antioxidant potential, as indicated by activity in the black and white rye extracts. Extracts prepared from black rye showed activity/percent inhibition as: AMRE (75.3%), MRE (66.8%), ERE (61.4%), ARE (49.2%) and BRE (22.8%). Extracts prepared from white rye showed activity/percent inhibition as: AMRE (69.2%), MRE (61.2%), ERE (54%), ARE (42.7%) and BRE (18.1%). Overall, water proved the most efficient solvent, followed by acidified methanol and methanol. Mishra et al. [39] studied cold water extracts prepared from rye cultivars, reporting 47.98% inhibition in extracts during a DPPH assay and 97.54% inhibition during an ABTS assay. The difference in percent inhibition may have been due to climatic conditions and geographical distributions. The TAC value indicates the potential of extracts to reduce from a Mo (VI) form to a Mo (V) form. The TAC of BR and WR was observed to be 0.9–6.8 mg AAE/g. The TAC for black rye extracts was observed as: WRE (6.8 mg AAE/g), ERE (1.8 mg AAE/g), MRE (3.9 mg AAE/g), ARE (1.7 mg AAE/g), AMRE (4.9 mg AAE/g) and BRE (0.9 mg AAE/g). Similarly, the TAC in white rye extracts was observed as: WRE (6.1 mg AAE/g), ERE (1.6 mg AAE/g), MRE (3.9 mg AAE/g), ARE (1.6 mg AAE/g), AMRE (4.5 mg AAE/g) and BRE (0.9 mg AAE/g). The FRAP of BR and WR extracts (WRE, ERE, MRE, ARE, AMRE and BRE) ranged from 1.1–8.2 mg FeSO_4_·7H_2_O equivalent/g. The lowest value was observed in white rye flour extracted with benzene (1.1 mg FeSO_4_·7H_2_O equivalent/g), whereas a higher FRAP was observed in black rye flour extracted with water (8.2 mg FeSO_4_·7H_2_O equivalent/g). The FRAP value of an extract represents its capability to reduce Fe^3+^ to Fe^2+^. Kulichova et al. [12] studied FRAP values of rye extracts that ranged from 2.27–5.36 mg Trolox equivalent/g. The RPA values of different rye extracts ranged from 0.7 to 8.7 mg QE/g. Kulichova et al. [12] reported a reducing power value of rye extracts in the range of 0.87–20.1 mg Trolox equivalent/g.

### 3.4. Relationship between Bioactive Compounds and Antioxidant Properties

**Figure 4:**
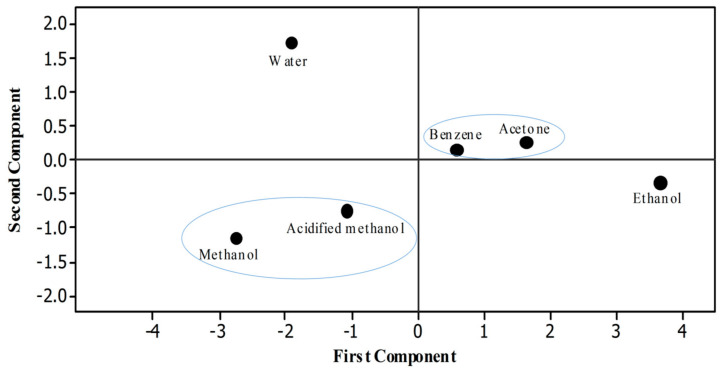
Score plot.

Statistical software Minitab was used to draw a score plot. Six different dots were recorded that indicated the efficacy of different solvents used to extract rye phenolics in the present study (Figure 4). To ease understanding, two different circles were drawn in which the properties or solvents that have positive relationships to each other are shown inside each circle. Pearson analysis was done to evaluate the correlation among different properties of rye extracts. Significant correlation was observed between the TPC and antioxidant assays (FRAP, r = 0.992 *p* < 0.01; TAC, r = 0.990; RPA, r = 0.969 *p* < 0.01; DPPH, r = 0.968 *p* < 0.01; ABTS, r = 0.882 *p* < 0.01) (Table 5).

### 3.5. DNA Damage Protection Potential (DDPP)

**Figure 5:**
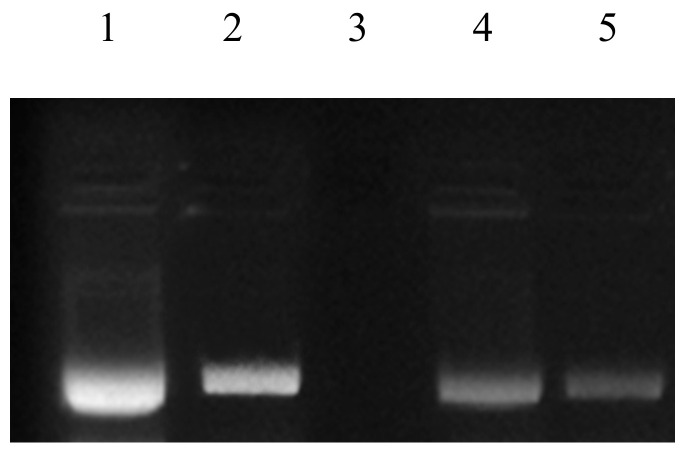
DNA damage protection activities in rye extracts against Fenton’s reagent. Lane 1: native pBR 322 plasmid DNA; Lane 2: DNA + Fenton’s reagent + Quercetin (mg/mL positive control); Lane 3: DNA + Fenton’s reagent; Lane 4: DNA + Fenton’s reagent + WRE (Black); Lane 5: DNA + Fenton’s reagent + WRE (White).

**Figure 6:**
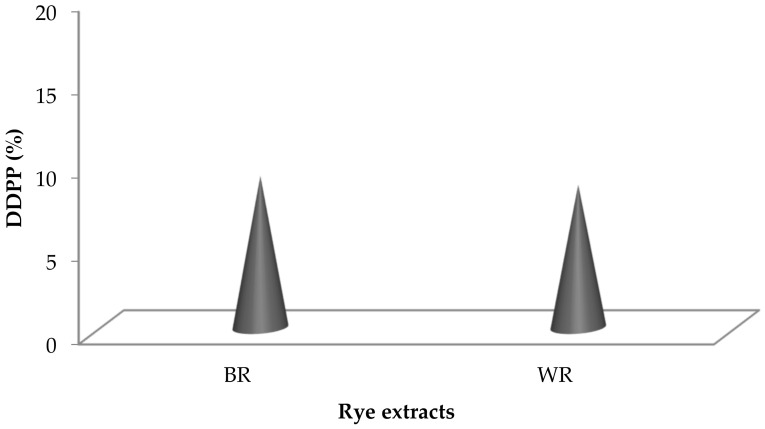
DDPP (%) in rye extracts.

Fenton’s reagent is extensively used as a DNA degrading agent [45,61] during DDPPA. Rye extracts prepared using water were subjected to DNA protection against the damaging activity of Fenton’s reagent on pBR322 (model DNA). Non-availability of bands during gel electrophoresis confirmed the damaging activity, whereas their appearance showed the presence of DDPP. The DDPP of rye extracts during electrophoresis is represented in Figure 5, shown by the appearance of bands in Lane 4 and Lane 5. Figure 6 represents the DDPP (%) of aqueous rye extracts. Published literature has also confirmed the presence of DDPP in extracts prepared from various botanical resources [45,62]. However, no report on DDPP in rye extracts has previously been published.

## 4. Conclusions

Comparison of different solvents (water, ethanol, methanol, acetone, acidified methanol and benzene) indicates water to be an efficient solvent for the liberation of phenolic compounds from selected rye cultivars. Maximum antioxidant properties were also observed in water extracts. The presence of specific phytochemicals in rye flour makes it a health-benefiting substrate that can be used in the preparation of various products for human use. For processing flour, the major solvent used at the domestic as well as the commercial scale is water. Efficacy of water for phenolic extraction from rye flour could prove important from an industrial point of view, as it is easily available and cost effective.

## Figures and Tables

**Table 1:** Phytochemicals, sugar, protein and alkaloids in different rye extracts.

Specific Phytochemicals/Tests	Solvents					
	Water	Ethanol	Methanol	Acidified Methanol	Acetone	Benzene
Phlabotannin	−	−	−	−	−	−
Coumarins	+	−	+	+	−	−
Flavonoids	−	−	−	+	−	+
Anthocyanins	−	−	−	−	−	−
Steroids	−	−	−	−	−	−
Molisch’s test	−	−	−	−	−	−
Fehling’s solution test	+	−	−	−	−	−
Benedict’s reagent test	+	−	−	+	−	−
Tannins	+	−	−	+	−	−
Protein	+	−	−	−	−	+
Saponins	+	+	+	+	+	+
Flavonon	−	−	−	−	−	−
Alkaloids	−	−	−	−	−	−

**Table 2:** Amount of specific compounds in aqueous extracts of different rye cultivars.

Cultivars	Specific Bioactive Compounds (mg/100 g)					
	Resorcinol	Catechol	Vanillin	Ferulic acid	Quercetin	Benzoic Acid
BR	70.4 ^b^ ± 0.11	120.4 ^b^ ± 0.08	5.5 ^b^ ± 0.23	1.52 ^a^ ± 0.15	4.68 ^a^ ± 0.12	5.3 ^a^ ± 0.18
WR	52 ^a^ ± 0.17	91.1 ^a^ ± 0.24	1.4 ^a^ ± 0.14	1.46 ^a^ ± 0.13	4.6 ^a^ ± 0.19	ND

Different letters (a,b) in column indicate that values are significantly different.

**Table 3:** Specific compounds in rye extracts and their health-benefiting features.

Specific Compounds	Chemical Formula	Health Benefits	Reference
Resorcinol	C_6_H_6_O_2_	Prevents acne formation, dermatitis, eczema, psoriasis, and other skin disorders; also used to treat corns, calluses, and warts	[51]
Catechol	C_6_H_6_O_2_	Anti-carcinogenic	[52]
Vanillin	C_8_H_8_O_3_	Anti-mutagenic, cosmetic and beverages industries	[53]
Ferulic acid	C_10_H_10_O_4_	Skin care product formulation	[51,54]
Quercetin	C_15_H_10_O_7_	Prevent heart diseases, cancer and blood sugar regulator	[55,56]
Benzoic acid	C_7_H_6_O_2_	Resolve skin related health issues, food preservative	[51,57]

**Table 4:** Antioxidant properties shown by rye extracts during different assays.

Solvents	DPPH (% Inhibition)		ABTS (% Inhibition)		TAC (mg AAE/g)		RPA (mg QE/g)		FRAP (mg FSE/g)	
	BR	WR	BR	WR	BR	WR	BR	WR	BR	WR
Ethanol	31.4 ^c^ ± 1.15	21.3 ^c^ ± 1.12	61.4 ^c^ ± 0.49	54.0 ^c^ ± 0.79	1.8 ^b^ ± 0.05	1.6 ^b^ ± 0.11	2.8 ^c^ ± 0.10	2.2 ^c^ ± 0.07	2.6 ^b^ ± 0.12	2.2 ^b^ ± 0.11
Methanol	43.5 ^d^ ± 0.90	38.7 ^d^ ± 1.06	66.8 ^d^ ± 0.41	61.2 ^d^ ± 0.62	3.9 ^c^ ± 0.03	3.9 ^c^ ± 0.08	4.1 ^d^ ± 0.05	3.9 ^d^ ± 0.04	5.4 ^c^ ± 0.15	4.1 ^c^ ± 0.18
Acetone	26.0 ^b^ ± 0.72	14.8 ^b^ ± 0.94	49.2 ^b^ ± 0.53	42.7 ^b^ ± 0.75	1.7 ^b^ ± 0.04	1.6 ^b^ ± 0.06	1.9 ^b^ ± 0.08	1.7 ^b^ ± 0.02	1.5 ^a^ ± 0.09	1.4 ^a^ ± 0.16
Acidified methanol	74.3 ^e^ ± 1.04	64.8 ^e^ ± 0.89	75.3 ^e^ ± 0.68	69.2 ^e^ ± 0.70	4.9 ^d^ ± 0.07	4.5 ^d^ ± 0.05	4.8 ^e^ ± 0.04	4.5 ^e^ ± 0.03	5.8 ^c^ ± 0.17	4.6 ^d^ ± 0.14
Benzene	14.1 ^a^ ± 1.08	10.7 ^a^ ± 1.05	22.8 ^a^ ± 0.96	18.1 ^a^ ± 0.83	0.9 ^a^ ± 0.09	0.9 ^a^ ± 0.07	0.9 ^a^ ± 0.02	0.7 ^a^ ± 0.09	1.2 ^a^ ± 0.11	1.1 ^a^ ± 0.13
Water	84 ^f^ ± 0.85	80.3 ^f^ ± 0.91	89.7 ^f^ ± 0.72	87.6 ^f^ ± 0.77	6.8 ^e^ ± 0.13	6.1 ^e^ ± 0.04	8.7 ^f^ ± 0.09	7.4 ^f^ ± 0.11	8.2 ^d^ ± 0.14	7.6 ^e^ ± 0.08

Different letters (a–f) in column indicate that values are significantly different.

**Table 5:** Correlation between different properties of rye extracts.

	TPC	CTC	DPPH	ABTS	TAC	RPA	FRAP
TPC	1	--	--	--	--	--	--
CTC	−0.424	1	--	--	--	--	--
DPPH	0.968 **	−0.380	1	--	--	--	--
ABTS	0.882 **	−0.045	0.915 **	1	--	--	--
TAC	0.990 **	−0.381	0.969 **	0.911 **	1	--	--
RPA	0.969 **	−0.263	0.948 **	0.925 **	0.976 **	1	--
FRAP	0.992 **	−0.352	0.981 **	0.914 **	0.992 **	0.982 **	1

** Correlation is significant at the 0.01 level.

## Data Availability

Data is contained within the article.

## Abbreviations

WRE: Water rye extract; ERE: Ethanol rye extract; MRE: Methanol rye extract; ARE: Acetone rye extract; AMRE: Acidified methanol rye extract; BRE: Benzene rye extract; GAE: Gallic acid equivalent; AAE: Ascorbic acid equivalent; QE: Quercetin equivalent; BR: Black rye; WR: White rye; FSE: Ferrous sulfate equivalent.
